# Persistence and course of mental health problems from childhood into adolescence: results of a 10-year longitudinal study

**DOI:** 10.1186/s40359-021-00535-4

**Published:** 2021-02-27

**Authors:** Max Supke, Caterina Ferling, Kurt Hahlweg, Wolfgang Schulz

**Affiliations:** Institute for Psychology, Technical University Brunswick, Humboldtstr. 33, 38106 Braunschweig, Germany

**Keywords:** Children, Adolescents, Persistence, Prevalence, Course, Longitudinal, Mental health problems

## Abstract

**Background:**

Mental health problems (MHP) in children and adolescents (CA) are common. This longitudinal study analyzed the prevalence, course, and persistence of MHP over 10 years from childhood into adolescence based on a sample from the *Future Family* project (*N* = 230).

**Methods:**

At the pre-assessment point the children were on average 5 (*SE* = 1) and the mothers 35 (*SE* = 5) years old. Descriptive methods, Chi^2^-tests, binary logistic regression, and different analytical approaches (number chains, transition probability) were used.

**Results:**

Approximately 24% of the CA suffered from borderline clinical or clinically relevant MHP. The largest proportion of the sample was *stable healthy* (70%), whereas 15% of the CA showed *chronic mentally ill*, 8% *transient,* 4% *negative* and 4% *positive* courses. The mental health of the mother proved to be a decisive predictor for *chronic mentally ill* courses. Short-term persistence rates ranged between 60 and 70% from one assessment point to the next one. On the other hand, long-term persistence rates (from childhood into adolescence) were lower (51–59%).

**Conclusion:**

One in seven children in this sample suffered from chronic MHP, while only one third of the CA in Germany with clinically relevant MHP take advantage of psychological or psychiatric care. Prevention programs should be considered as an effective and economic approach to reduce childhood suffering in Germany.

**Supplementary Information:**

The online version contains supplementary material available at 10.1186/s40359-021-00535-4.

## Background

Mental health problems (MHP) in childhood and adolescence are a worldwide health challenge of the twenty-first century. Many children and adolescents (CA) suffer from MHP during their development. In order to develop effective prevention and intervention programs, a comprehensive knowledge of the prevalence, development and persistence of MHP is indispensable.

The worldwide prevalence of mental disorders in CA is approximately 13.4% according to an international meta-analysis, which analyzed 41 studies and 87.742 CA in 27 countries from every world region [1]. In a German meta-analysis including 33 studies and 77.978 CA Barkmann and Schulte-Markwort [2] reported an average MHP prevalence rate of 17.6%. Overall, the number of mental disorders seems to be slightly higher in Germany compared to the worldwide prevalence. On the other hand, the representative German Health Interview and Examination Survey for Children and Adolescents (KiGGs) reported a significant reduction of child MHP in the last years. While in the baseline study (2003–2006) 19.9% of the CA suffered from MHP, the second wave 10 years later showed a significantly lower prevalence of 16.9% [3].

While many cross-sectional studies focus on the prevalence of MHP in CA, the evidence on the course and persistence needs improvement. Especially in Germany the number of longitudinal studies is insufficient [4, 5].

For instance, Esser, Ihle, Schmidt and Blanz [4] assigned 80% of the CA to the *stable healthy*-type (clinically relevant values at none or only one out of four assessment points) and 10% to the *chronical mentally ill-*type (three or four out of four assessment points were clinically relevant). This distribution coincides with the results of the BELLA-study by Ravens-Sieberer et al. [5] reporting a rate of 74% *stable healthy* CA, 16% *remitted*, 3% *persistent* and 7% *acute or recurrent* MHP. Based on the current state of research, it can be assumed that the majority of CA in Germany are psychologically *stable healthy*, while one in ten children suffers from *chronic* or *recurrent* MHP.

With regard to the general stability independent of diagnoses in CA, a persistence rate of about 50% can be assumed for German CA [4–6]. This is consistent with the review by Ihle and Esser [7], in which consistently high persistence rates of usually more than 50% were reported across different ages of CA. Ravens-Sieberer et al. [5] also described a high risk of childhood and adolescent MHP persisting into adulthood. Persistence rates of 50% were found in the first and second year after the BELLA baseline survey. After 6 years, 32% of the CA who showed MHP in the baseline survey were still at risk. However, it should be noted that different definitions and methodological approaches were often used to analyze the persistence. For this reason, the results are often not completely comparable.

With respect to Internalizing (e.g., Depression) and Externalizing (e.g., ADHD) disorders in general, the research results are somewhat ambiguous. However, studies seem to predict a higher stability of Externalizing MHP predominate in Germany [4, 7]. Various individual diagnoses have also been examined in numerous studies with regard to their course in CA. There are mainly consistent results on the course of depressive disorders in CA indicating a high persistence and an increase in their prevalence with age [8, 9]. Regarding the course of anxiety disorders, an increase from childhood to adolescence should be expected. However, it should be noted that different anxiety disorders have diverse courses [8]. The number of CA with ADHD decreases in the course of development, while the course is often described as favorable [4, 8, 10]. On the other hand, Ihle and Esser [7] assume an unfavorable course, while Hölling et al. [11] found a temporal stability of ADHD.

*Sex-* and *age-specific* differences play a major role when considering MHP and their persistence. Childhood MHP appear to be both more common and more persistent in boys than girls. For instance, higher prevalence rates are often found for boys up to the age of 11–14 years, while the prevalence rates of MHP in adolescence seem to converge for both genders [3, 6, 7, 9, 12]. This could be explained by the finding that in adolescence girls seem to show a greater increase in the prevalence of MHP [12] and a higher stability [6].

On the one hand, some risk factors for the development of the children seem to play a greater role in early childhood. These include, for example, mother-infant interactions (e.g., [13]). On the other hand, other factors like peer relationships (e.g., [14]) appear to be more relevant for MHP in adolescence, whereas for example dysfunctional parenting seems to play a significant role from early childhood until adolescence [15]. Furthermore, transgenerational mechanisms of MHP within a family should also be considered [e.g., 16]. With regard to risk factors for the persistence of MHP, one study found that maleness, one-carer households, poor physical health and low cognitive abilities of the child were significant predictors for the continuity of psychopathology between mid- to late childhood. Caregiver depression was thereby the most important familial variable [17].

First, it should be considered that there are only a few longitudinal studies which explore the course of MHP in CA overall and especially in Germany. Due to different definitions and assessment methods (interviews, questionnaires, self-report vs. parent-report) of MHP and their persistence the comparability of the studies is limited. Furthermore, often only short periods of time and single age groups were examined. The following report from a 10-year longitudinal study takes these shortcomings into account and addresses the lack of available studies.

## The present study

The central research questions this study aims to answer are: How high is the prevalence rate of MHP in CA in our sample? We expected frequencies of MHP similar to those reported in the German meta-analysis (17.6%) [2].

How high is the proportion of chronic MHP? Based on the state of research, we expected that about 10% of the children and adolescents are affected by persistent MHP (*chronic mentally ill*).

How high is the persistence of MHP in CA? Furthermore, differences between Internalizing and Externalizing MHP as well as sex-specific aspects will be examined.

The persistence should be around 50%. Externalizing MHP should be more persistent within child development than Internalizing MHP. The prevalence and persistence rates should be higher for boys until adolescence.

Finally, risk and protective factors for the course of MHP in childhood and adolescence will be explored. We expected that *chronic mentally ill* children are male, have a lower socio-economic status, their parents show worse mental health, and the parenting competence is more problematic. On the other hand, the child’s intelligence could be a protective factor [17].

## Method

The data were collected as a part of the DFG-projects (German Research Foundation) *Future Family I* (FF I) [18] and *Future Family III* (FF III) [19] in Germany. The FF I-study is a randomized controlled study in which the effectiveness of the parenting training *Positive Parenting Program* (Triple P) [20] was examined. This study was based on a longitudinal design with five assessment points: Pre-assessment and follow-up after 1, 2, 3 and 4 years (= FU1, FU2, FU3 and FU4). The FF III-study explored the effectiveness of Triple P and the prediction of MHP in adolescence after 10 years (FU10) considering risk and protective factors of kindergarten age (Pre).

## Recruitment and characteristics of the sample

All 33 kindergartens in Brunswick (Germany) were informed about the FF I-project, 23 expressed interest in participating. From these, 17 kindergartens were randomly selected according to their number of children and their catchment area parallelized and then randomized. Criteria for the participation of families in the project were the age of the child (2.6–6 years), the care of the child in the kindergarten as well as a basic understanding of the German language. A total of *N* = 280 families were recruited in 2001/2002 (*M*_mothers_ = 35 years, *SD* = 5; *M*_fathers_ = 39 years*, SD* = 6; *M*_children_ = 4 years, *SD* = 1; 51% boys).

For the 10 year-follow-up survey, 249 families could still be assessed (retention rate: 89%). Nineteen families were excluded from the analyses due to missing data (final *N* = 230). At this assessment point, the average age of adolescents was 14 (*SD* = 1) years. Further sample characteristics: 52% boys; education of mothers: no school graduation/Certificate of Secondary Education (Hauptschulabschluss): 10%, General Certificate of Secondary Education (Realschulabschluss): 33%, High School (Abitur): 57% (fathers: 13%/23%/64%); 57% of the adolescents attended grammar school. Every sixth mother was a single parent, and every tenth family had a migration background (see also Additional file 1: Table 1).

## Procedure and measures

The data were acquired using a combination of interviews (e.g., sociodemographic data) and standardized questionnaires. The interviews with the parents and adolescents took place parallel in separate rooms, usually during a home visit.

MHP of the children as well as other characteristics were assessed by the mothers and fathers. Due to the substantial number of missing values among the questionnaires filled out by fathers (22–29%), only the data of mothers were analyzed. The data of the children and parents were collected on the one hand from pre-school (3–5 years; Pre/FU1/FU2) to school age (6–8 years; FU3/FU4) and on the other hand once in adolescence (Range of the adolescent’s age: 13–16 years, FU10) [15].

The study was conducted according to the principles stated in the Declaration of Helsinki (59th WMA General Assembly, Seoul, 2008). The research project received ethical approval by the ethics committee of the German Psychological Society (DGPs; identification number: WS 12_2010).

When planning our study, we followed Belsky’s classic “process of parenting model” [15, 21]. He proposed that parenting is multiply determined and influenced by characteristics of a) the parent (e.g., depression, personality, marital relation), b) the child (e.g., temperament, negative emotionality) and c) the family’s social context (see also [22, 23]). The model has become a classic with over 4400 citations in the literature [15]. The assessment instruments used were selected according to the model from 1984.

### Child behavior checklist (Pre, FU2, FU3, FU4, FU10)

The project followed the concept of evidence based multimodal assessment [24]. We decided to use the *Achenbach System of Empirically Based Assessment* (ASEBA [25]). The ASEBA assess in a developmentally appropriate way a) via parent’s report on the Child Behavior Checklist (CBCL) for ages 6 to 18, b) teacher’s report on the Teacher’s Report Form (TRF), and c) adolescent’s report on the Youth Self Report YSR for ages 11 to 18. These forms also assess problems, competencies, as well as adaptive functioning and are particularly suited for longitudinal research.

For this study, we used the German version of the CBCL (Pre: CBCL 1½–5 [26]; FU2-FU10: CBCL 4–18 [27]) to assess MHP in children and adolescents. Parents were presented 110–118 items and asked to rate the frequency of certain behaviors of their child on a 3-point Likert scale (0 [*not true*]; 1 [*somewhat or sometimes true*]; 2 [*very true or often true*]). Based on the CBCL, a total score as well as scores for Internalizing and Externalizing MHP can be formed. Higher scores indicate more MHP. The mean internal consistencies over the different assessment points in our sample can be rated as good (α = 0.84–0.95).

### Mental health problems of the mother (Pre)

MHP of the mother were assessed using the German version [28] of the *Depression Anxiety Stress Scales* (DASS) [29].

This self-report questionnaire consists of 42 items that are related to the three scales of depressive mood, anxiety, and stress. The scores of the three subscales can be summarized to a total value. Higher scores indicate more maternal MHP. The internal consistency in our sample can be rated as excellent (α = 0.94).

### Dysfunctional parenting behavior (Pre)

Maternal dysfunctional parenting behavior was assessed using the German version (EFB) [30] of the *Parenting Scale* [31]. The 35 items of the self-report questionnaire can be summarized to a total score, whereas a higher score indicates more use of dysfunctional parenting practices. The internal consistency in our sample can be rated as good (α = 0.87).

### Intelligence of the child (Pre)

The intelligence of the children was assessed using the German version (K-ABC) [32] of the *Kaufman Assessment Battery for Children* (K-ABC) [33]. The instrument consists of 16 subtests, which vary according to the age of the child. The results of the tests can be combined into four subscales: mental, sequential, and simultaneous processing as well as achievement. The intellectual abilities subscale (mental processing) was used for this analysis, whereas higher values indicate higher intellectual abilities.

## Determination of course-types and persistence rates

This study analyzed the persistence of MHP using two methodically different approaches. First, a distinction was made between the absence (0) and presence (1) of MHP at every assessment point based on the age-specific T-scores of the maternal CBCL-scales. Borderline clinical (T = 60–63) and clinically relevant scores (T ≥ 64) were combined and used as an indicator for MHP [26, 27]. This resulted in a sequence of numbers for each child over five assessment points, e.g., 0 1 0 1 1.

In the following paragraph, the five different classes of course-types are described and an example is listed: (a) *chronic mentally ill* (at least four out of five assessment points were clinically relevant: 1 1 1 1 0); (b) *stable healthy* (at least at four out of five assessment points MHP were negative: 0 0 1 0 0); (c) *positive course* (at least at the last two assessment points MHP were negative: 1 1 1 0 0); (d) *negative course* (at least at the last two assessment points MHP were present: 0 1 0 1 1); (e) *transient course* (at least two changes from negative to present, while the last two assessment points were different: 0 0 1 0 1).

The second approach was based on the method of calculating transition probability as used in the study by Esser and colleagues [4]. The authors analyzed how many CA still showed MHP or recovered from MHP from one assessment point to the next one. Additionally, they also looked at how many of the healthy CA stayed healthy or developed MHP, whereby the observations periods were 5 and 7 years. Contingency tables and figures were used for calculation and presentation.

## Statistical analyses

The analyses were mainly descriptive using the two approaches described before. Complete CBCL-data were available from all families at the pre-assessment point. Missing values at FU2 (*n* = 7), FU3 (*n* = 6) and FU4 (*n* = 14) were imputed with the MICE package (multivariate imputation by chained equations) [34] using the statistical software R [35]. To determine protective and risk factors of the persistence, binary logistic regression models were computed.

## Results

### Prevalence of mental health problems in our sample in childhood and adolescence (CA)

The prevalence rates in our sample differentiated by assessment points, type of MHP, and sex (boys *n* = 119, girls *n* = 111) are shown in Table 1. The prevalence for the CBCL-Total Score ranged between 14.8% and 28.7% with a mean rate over all assessment points of *M*_Pre-FU10_ = 23.6%. The prevalence rates for Internalizing MHP ranged between 17.0% and 28.3% (*M*_Pre-FU10_: 24.3%), for Externalizing MHP between 14.8% and 29.1% (*M*_Pre-FU10_: 22.9%). While Externalizing behavior was most frequently found in childhood (FU2), Internalizing behavior occurred most often in adolescence (FU10).

**Table 1:** Prevalence of MHP in percentages based on the maternal CBCL-ratings over all assessment points (N = 230). Clinical scores (T ≥ 64) in brackets

	Pre	FU2	FU3	FU4	FU10	Mean
Total score	14.8	28.7 (16.1)	25.7 (15.2)	22.2 (14.3)	26.5 (15.7)	23.6 (15.3)
Girls	12.6	27	26.1	20.7	23.4	22
Boys	16.8	30.3	25.2	23.5	29.4	25
Internalizing	17	26.5 (15.2)	26.5 (13.0)	23.0 (13.0)	28.3 (15.7)	24.3 (14.2)
Girls	16.2	27	28.8	25.2	25.2	24.5
Boys	17.6	26.1	24.4	21	31.1	24
Externalizing	14.8	29.1 (16.5)	23.9 (17.8)	22.6 (13.5)	23.9 (12.6)	22.9 (15.1)
Girls	11.7	27	24.3	21.6	21.6	21.2
Boys	17.6	31.1	23.5	23.5	26.1	24.4

When calculating the mean value for the clinically relevant frequencies, the pre- and 1-year assessment points were omitted due to methodological problems (CBCL 1.5–5: no German norms available)

Regarding Internalizing MHP, there were no differences between boys and girls at any assessment point (girls: 24.5% vs. boys: 24.0%). Boys were affected slightly more by Externalizing MHP than girls (girls: 21.2% vs. boys: 24.4%). Overall, there were no significant sex differences according to Chi^2^-tests.

In general, the prevalence rates of MHP in our sample seem to decrease as the child gets older, whereas in adolescence MHP, in particular Internalizing MHP, occur more frequently.

### Persistence rates—method 1: course-types

The *stable healthy* type was the most common (69–71%), while similar frequencies resulted for the three MHP categories: Total Score, Internalizing and Externalizing behavior. Second most frequently was the *chronic mentally ill* type (14–15%). The course-types *positive* (3–4%), *negative* (3–4%) and *transient* (8–10%) occurred less frequently (Table 2).

**Table 2:** Distribution of course-types and sex differences (N = 230, in %)

	Stable healthy	Chronic mentally ill	Positive course	Negative course	Transient course	Boys versus girls (Chi^2^-test)
Total score	69.1	15.2	3.9	3.5	8.3	*χ*^2^ = 2.6, *df* = 4
Girls	70.3	15.3	5.4	2.7	6.3	*p* = .622
Boys	68.1	15.1	2.5	4.2	10.1	*V* = .11
Internalizing	70.9	13.9	3	2.6	9.6	*χ*^2^ = 4.0, *df* = 4
Girls	68.5	12.6	2.7	2.7	13.5	*p* = .403
Boys	73.1	15.1	3.4	2.5	5.9	*V* = .13
Externalizing	70.9	13.5	3.5	3.5	8.7	*χ*^2^ = 9.6, *df* = 4
Girls	73.9	13.5	0.9	0.9	10.8	*p* = .048*
Boys	68.1	13.4	5.9	5.9	6.7	*V* = .20

*p < .05

Only Externalizing MHP showed significant sex-specific differences (*χ*^2^ = 9.6, *df* = 4, *p* = 0.048; *V* = 0.20) in their distributions: Boys were significantly more often assigned to the *positive* (*χ*^2^ = 4.2, *df* = 1, *p* = 0.039; *V* = 0.14) or *negative course*s (*χ*^2^ = 4.2, *df* = 1, *p* = 0.039; *V* = 0.14) compared to girls.

### Persistence rates—method 2: transition probabilities

**Fig. 1:**
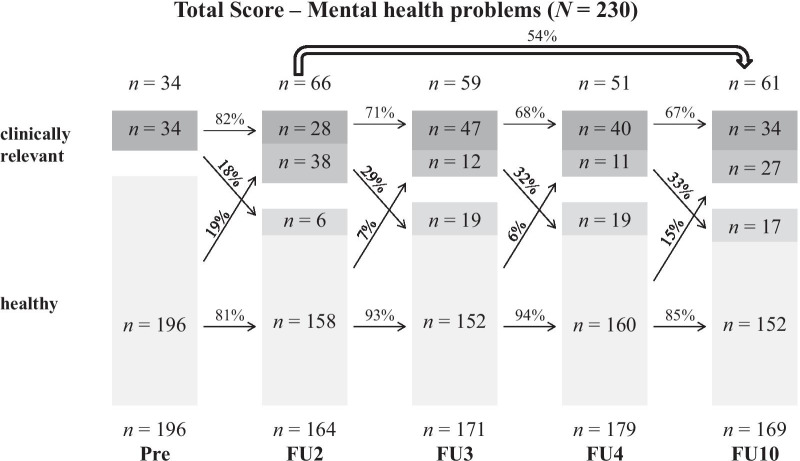
Persistence of MHP based on maternal CBCL-total scores over the course of 10 years

**Fig. 2:**
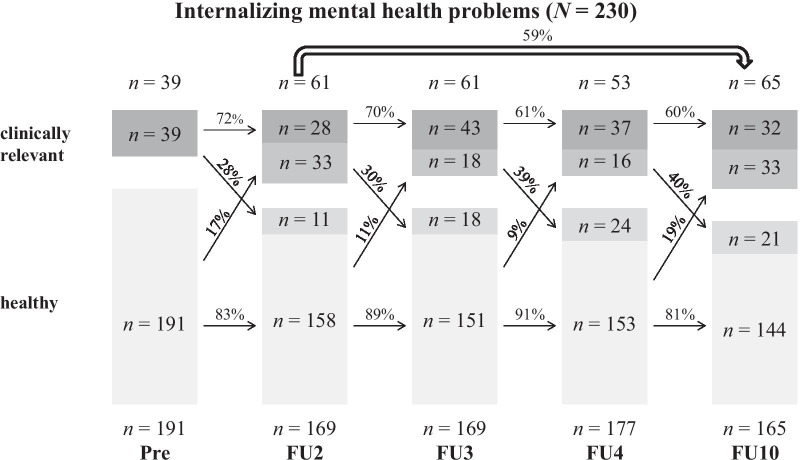
Persistence of internalizing MHP based on maternal CBCL-ratings over the course of 10 years

**Fig. 3:**
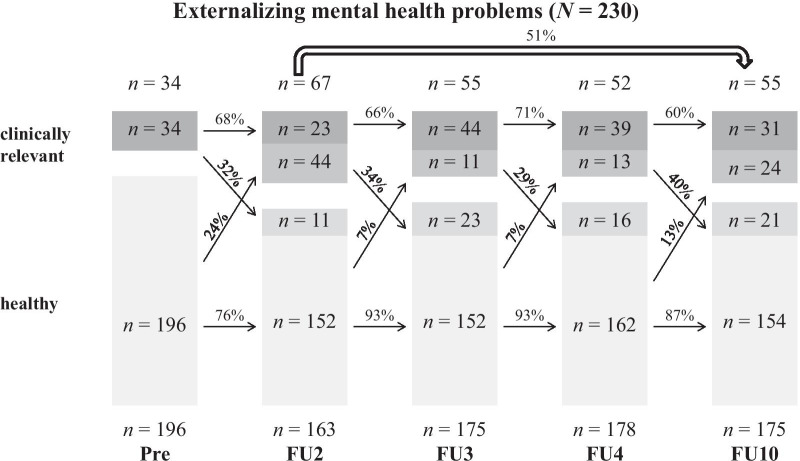
Persistence of externalizing MHP based on maternal CBCL-ratings over the course of 10 years

Figures 1, 2 and 3 show the persistence rates of all three analyzed categories. In terms of the Total Score (Fig. 1) of the 34 children with MHP at the pre-assessment point, 82% continued to suffer from MHP, while 18% were healthy at FU2. On the other hand, of the 196 healthy children at pre, 81% remained healthy, while 19% developed clinically relevant MHP.

In total, high persistence rates (60–82%) were found between the assessment points. Both Internalizing and Externalizing MHP were similar in their short-term persistence (from one assessment point to the next one) in early childhood. Additionally, there was a tendency for the persistence to decrease with age.

Furthermore, the long-term persistence rates from FU2 (age: 6) to FU10 (age: 14) were calculated. With this method, lower persistence rates were found (Total Score: 54%). Internalizing MHP (59%) showed slightly higher persistence rates compared to Externalizing MHP (51%).

Detailed figures and tables for sex-specific courses of boys and girls can be found in Additional file 1. The Total Score showed similar short-term persistence rates for boys and girls. Regarding Internalizing and Externalizing MHP, boys and girls showed a relatively similar persistence until the start of adolescence. At the transition into puberty, the short-term persistence was significantly higher in boys for both categories (Internalizing: girls: 43% vs. boys: 80%; Externalizing: girls: 50% vs. boys: 68%).

While for the Total Score (girls: 47% vs. boys: 59%) and Internalizing MHP (girls: 50% vs. boys: 68%) boys showed a higher long-term persistence, girls’ long-term persistence for Externalizing MHP was slightly higher (girls: 53% vs. boys: 49%).

### Risk and protective factors: *stable healthy* versus *chronic mentally ill*

Table 3 shows the results of the three logistic regression models (Total Score, Internalizing, Externalizing) used to predict the differences between the *stable healthy* and *chronic mentally ill* courses. While child sex and IQ, mother’s education, and mother’s parenting at pre-assessment were non-significant predictors, maternal mental health proved to be the most important predictor for all three categories (*B*_total_ = 0.05, *p* = 0.004, *OR*_total_ = 1.05; *B*_int_ = 0.04, *p* = 0.011, *OR*_int_ = 1.04; *B*_ext_ = 0.05, *p* = 0.003, *OR*_ext_ = 1.05;). The higher the DASS-score of the mother at pre, the higher the odds that the child will have a *chronical mentally ill* course. For Internalizing MHP, low family income was another significant predictor *(B* = − 0.19, *p* = 0.015, *OR* = 0.83). The models explained 22–24% of the variance.

**Table 3:** Results of the logistic regression to differ between stable healthy und chronic mentally ill courses. Only variables from the pre-assessment point were used

	Total Score			Internalizing			Externalizing		
	*B*	*p*	*OR*	*B*	*p*	*OR*	*B*	*p*	*OR*
Sex-child	− .05	.907	0.95	.44	.330	1.55	.23	.602	1.25
Education-mother	.18	.690	1.20	− .35	.455	0.70	.61	.206	1.85
Income-household	− .14	.090	0.87	− **.19**	.015*	**0.83**	− .12	.149	0.89
Mental health (DASS)-mother	**.05**	.004**	**1.05**	**.04**	.011*	**1.04**	**.05**	.003**	**1.05**
Parenting (EFB)-mother	.54	.192	1.72	.27	.522	1.31	.40	.340	1.50
Intelligence (K-ABC)-child	− .04	.066	0.97	− .01	.784	0.99	− .03	.126	0.97

Significant results are in bold

Reference categories: sex: girls (0)/boys (1) − education: lower (0)/higher education (1). Model Total Score: *n* = 182, *χ*^2^ = 31, *p* < *.*000, Nagelkerkes *R*^*2*^ = .26, correct prediction: 85%. Model Internalizing: *n* = 183, *χ*^2^ = 26, *p* < *.*000, Nagelkerkes *R*^2^ = .23, correct prediction: 86%. Model Externalizing: *n* = 185, *χ*^2^ = 26, *p* < *.*000, Nagelkerkes *R*^2^ = .22, correct prediction: 86%

*p < .05; **p < .01

## Discussion

In Germany, studies focusing on the course of MHP from childhood into adolescence are insufficient. For this reason, this study within the Future Family project tried to examine how frequent and stable MHP are from kindergarten age to adolescence over the course of 10 years. We expected that about 18% of children are affected by MHP and that MHP have a persistence rate of around 50%. Every tenth child should show a *chronic mentally ill* course.

### Prevalence of mental health problems (MHP) in our sample in childhood and adolescence

Approximately 24% of the sample showed borderline clinical or clinically relevant MHP. Internalizing MHP seem to occur slightly more frequently than Externalizing MHP. These rates are similar to the results of the KiGGS-study, which found 20% borderline and clinically relevant MHP [11]. If only the clinically relevant rates are considered, a prevalence rate of about 15% was found, which is slightly higher than the worldwide (13%) [1] and slightly lower than the prevalence rate reported in Germany (17–18%) [2, 3]. Furthermore, when observing the course of MHP, it is apparent that the prevalence rates decrease overall with increasing age in childhood, while a large rise, especially in Internalizing MHP, was found in puberty. Thus, the onset of adolescence seems to be a particularly vulnerable time in child development.

Non-significant differences in prevalence rates between boys and girls were found. Nevertheless, there was a great increase in Internalizing MHP for boys during puberty. The assumption that boys in Germany show higher prevalence rates than girls until puberty, therefore, could not be confirmed [3, 5, 7, 9, 12].

### Persistence of mental health problems in childhood and adolescence

Regarding the first method using course-types, we found similar results for all three analyzed categories. Approximately 70% of the CA never showed any or only once MHP, whereas 14–15% were *chronic mentally ill*. While we found comparable frequencies to the KiGGS-sample (74%) [5] for the *stable healthy* group, our sample showed slightly more *chronic mentally ill* courses than previously assumed by Esser et al. (10%) [4]. Furthermore, the same number of children (3–4%) experienced a *positive* or *negative* course. About 8–10% of our sample showed *transient* courses meaning that their MHP were only clinically relevant at some assessment points. This could provide evidence that a greater proportion of CA suffer from recurrent MHP. Referring to sex-specific aspects, significant different distributions were only found for Externalizing MHP. Boys showed significantly more often a *negative* or *positive* course, whereby both course-types were represented equally frequently.

Our second approach was methodologically oriented to Esser et al. [4] and their transition probabilities. In the following results, a distinction between short- (from one assessment point to the next one) and long-term (from FU2 to FU10 = from childhood into adolescence) persistence should be made. Regarding the short-term persistence, similar percentages of mostly around 60–70% were found for the Total Score as well as Internalizing and Externalizing MHP. Our results differ from the BELLA-study [5], which reported persistence values from 30 to 50%. The reason for this can be found in the method. In the BELLA-study CA from 7 to 17 years formed the baseline sample, whereas in this study the course of kindergarten children from 3 to 5 years was analyzed. Accordingly, in this sample the course of MHP is considered in a sample of children with similar age. Another explanation for these high persistence values is the combination of borderline clinical and clinically relevant values leading to higher prevalence rates. Furthermore, hints for a decreasing tendency with age was found, which was also visible in the BELLA-study. The short-term persistence should be rated as high, taking into account the short time intervals of only 1 year between most assessment points.

Long-term persistence rates from childhood to adolescence were highest for Internalizing MHP (59%), while the Total Score (54%) and Externalizing MHP (51%) showed slightly lower rates. Overall, these results are in line with the findings from Esser and colleagues [4] as well as Ihle and Esser [9], reporting persistence rates of more than 50%. Contrary to our assumption, Internalizing MHP were more stable than Externalizing MHP. When looking at the sex-specific differences, particularly clinically relevant values in childhood reported by boys showed high persistence rates up to puberty for all three categories. While the long-term persistence rates from girls were around 50%, boys MHP were more stable, both in the Total Score (59%) and Internalizing MHP (68%).

For the prediction of chronic courses, MHP of the mother in particular proved to be a decisive predictor, whereas child’s intelligence, mother’s education, dysfunctional parenting, and sex showed no significant influence in these models. The finding that MHP of the mother contribute to the continuity of pathology in their children is consistent with the results of O’Connor and colleagues [17] identifying depression in the primary caregiver as the only significant familial variable predicting the persistence. On the other hand, the sex and cognitive abilities of the child as well as socioeconomic aspects showed significant results in their study, which is in contrast to our results. To prevent *chronic mentally ill* courses in children, the mental health of the mother could be a significant starting point for interventions.

### Strengths and limitations

On the positive side, data were collected from kindergarten children up to adolescence, with three different categories of MHP being analyzed simultaneously. Furthermore, ideas for the analysis of persistence in the form of number chains (0 0 0 1 1) and the distinction between short- and long-term persistence were presented.

On the other hand, several limitations should be discussed. First, only the mother’s perspective was analyzed. Second, since the sample is mainly from Brunswick and comes from the higher social class, which could lead to lower MHP [3], the generalizability of the results is limited. Third, for the CBCL1½—5 no German norms are available which is why the American norms were used for the pre-assessment point. Due to the more liberal American norms at pre, the reported prevalence rates were significantly lower and an increase in MHP at FU2, when German norms were used, is noticeable. This inevitably had a direct impact on the analysis of the number sequences and the persistence rates. Additionally, a huge proportion (54%) of the parents in our sample participated in the Triple P [20], a parenting program reducing dysfunctional parenting and preventing MHP in CA leading to altered and potentially lower prevalence and persistence rates. Taking these aspects into account, it can be assumed that the number of clinically relevant MHP should be even higher than reported in this study. Finally, the co-occurrence of Internalizing and Externalizing MHP was not further analyzed, while it could play an important role in child development and the persistence of MHP [36].

## Conclusion

Mental Health problems (MHP) are not uncommon in CA, with one in seven children in this sample suffering from chronic MHP. Only around 30% of the CA in Germany with clinically relevant MHP take advantage of psychological or psychiatric care [37].

If a child did not suffer from clinically relevant MHP at one assessment point, there was a high chance that they were healthy at the next one as well. This reflects the importance of effective prevention and intervention programs for CA. In particular prevention programs in early childhood are a method of early prevention for long-term chronic MHP, while they are an economic option reaching a large number of families with a possible high rate of return [38, 39]. The most important argument, however, should be that the affected CA are potentially spared many years of suffering from MHP, which have a high chance of extending into adulthood as well.

In conclusion, it can be said that in this study, new insights into the course of MHP from childhood into adolescence were found, and the deficient study situation in Germany could be improved, however, many more studies are necessary to reach strong conclusions.

## Supplementary Information

**Additional file 1**. Sample characteristics and sex-specific persistence rates. Additional file 1 contains a table with the sample characteristics as well as tables and figures showing the persistence rates of boys and girls.

## Data Availability

The datasets generated and/or analyzed during the current study are not publicly available as they contain sensitive material. Furthermore, it is a longitudinal study with several assessment points, so that the data could possibly be used to draw conclusions about individuals. The questionnaires used can be found in the corresponding references.

## Abbreviations

CA: Children and adolescents
FF: Future family
FU: Follow-up
MHP: Mental health problems
