# Supplementary material for: Persistence and course of mental health problems from childhood into adolescence: results of a 10-year longitudinal study
Source: BMC Psychol. 2021 Feb 27;9:38. doi: 10.1186/s40359-021-00535-4 (PMC7912888; doi:10.1186/s40359-021-00535-4)
Supplement: Supplementary file 1 — Additional file 1. Sample characteristics and sex-specific persistence rates. Additional file 1 contains a table with the sample characteristics as well as tables and figures showing the persistence rates of boys and girls. [file 40359_2021_535_MOESM1_ESM.pptx]

## Slide 1
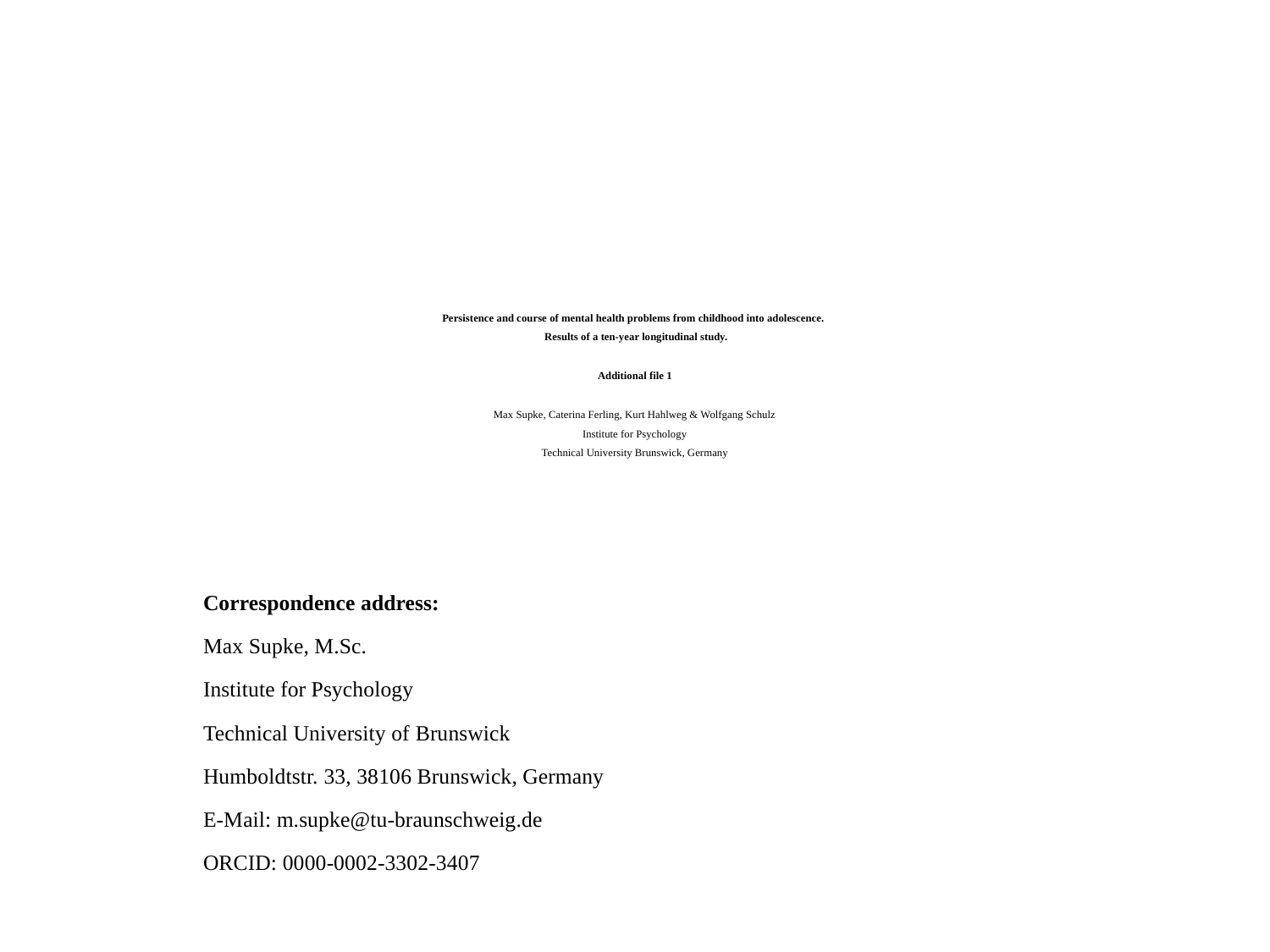

# Persistence and course of mental health problems from childhood into adolescence. Results of a ten-year longitudinal study. Additional file 1   Max Supke, Caterina Ferling, Kurt Hahlweg & Wolfgang Schulz Institute for Psychology Technical University Brunswick, Germany
Correspondence address:
Max Supke, M.Sc.
Institute for Psychology
Technical University of Brunswick
Humboldtstr. 33, 38106 Brunswick, Germany
E-Mail: m.supke@tu-braunschweig.de
ORCID: 0000-0002-3302-3407

## Slide 2
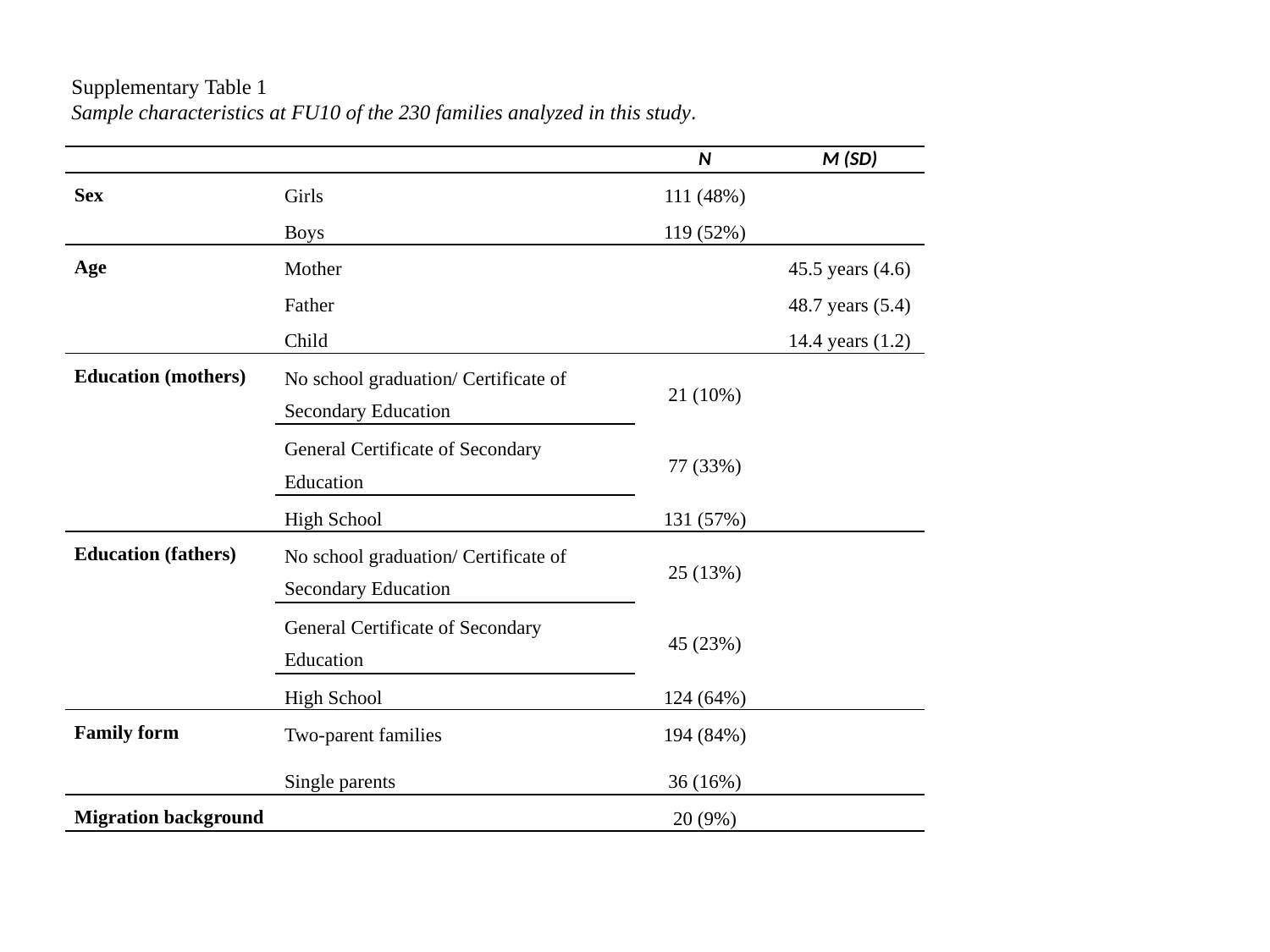

Supplementary Table 1
Sample characteristics at FU10 of the 230 families analyzed in this study.
| | | N | M (SD) |
| --- | --- | --- | --- |
| Sex | Girls | 111 (48%) | |
| | Boys | 119 (52%) | |
| Age | Mother | | 45.5 years (4.6) |
| | Father | | 48.7 years (5.4) |
| | Child | | 14.4 years (1.2) |
| Education (mothers) | No school graduation/ Certificate of Secondary Education | 21 (10%) | |
| | General Certificate of Secondary Education | 77 (33%) | |
| | High School | 131 (57%) | |
| Education (fathers) | No school graduation/ Certificate of Secondary Education | 25 (13%) | |
| | General Certificate of Secondary Education | 45 (23%) | |
| | High School | 124 (64%) | |
| Family form | Two-parent families Single parents | 194 (84%) 36 (16%) | |
| Migration background | | 20 (9%) | |

## Slide 3
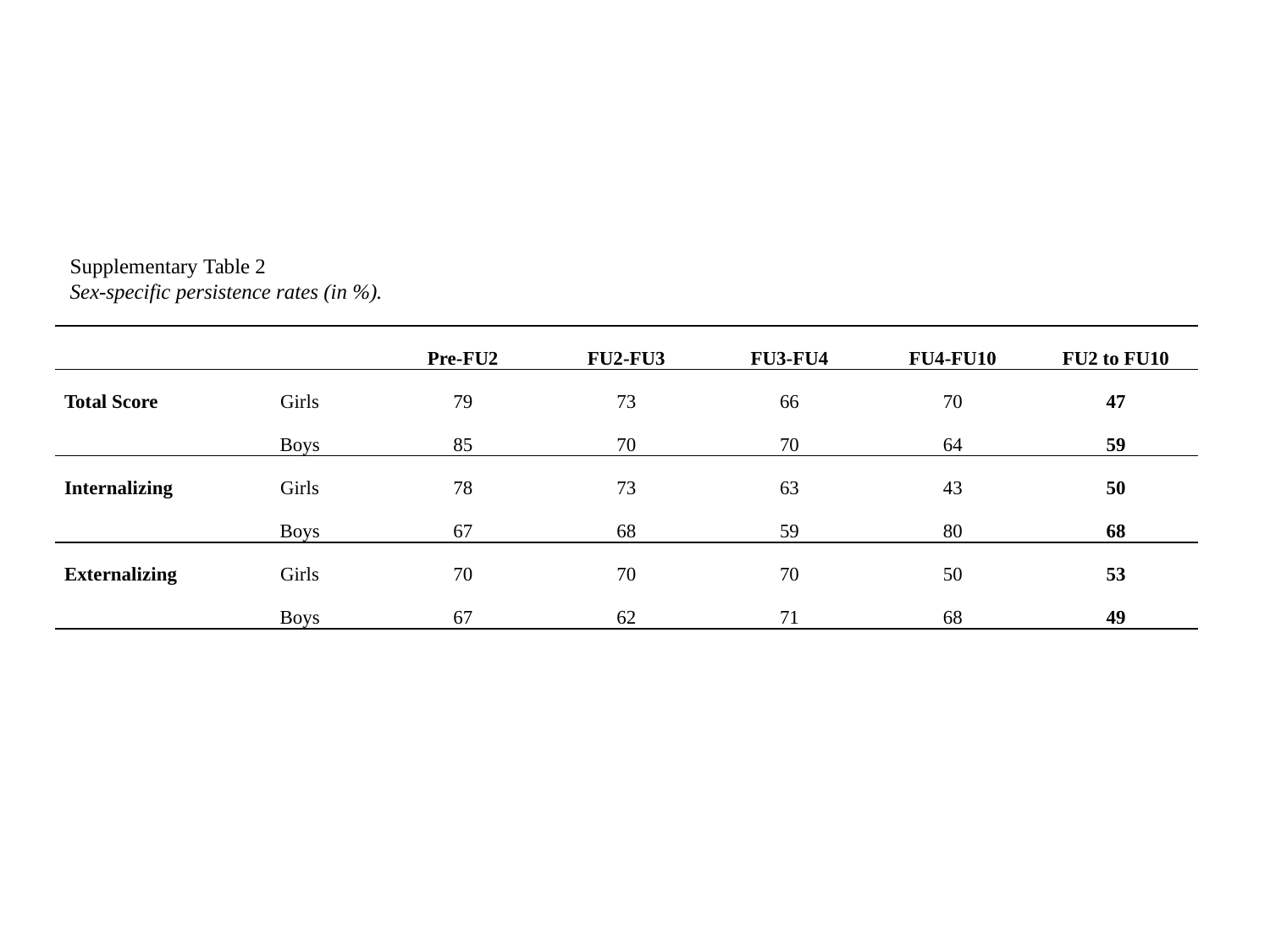

Supplementary Table 2
Sex-specific persistence rates (in %).
| | | Pre-FU2 | FU2-FU3 | FU3-FU4 | FU4-FU10 | FU2 to FU10 |
| --- | --- | --- | --- | --- | --- | --- |
| Total Score | Girls | 79 | 73 | 66 | 70 | 47 |
| | Boys | 85 | 70 | 70 | 64 | 59 |
| Internalizing | Girls | 78 | 73 | 63 | 43 | 50 |
| | Boys | 67 | 68 | 59 | 80 | 68 |
| Externalizing | Girls | 70 | 70 | 70 | 50 | 53 |
| | Boys | 67 | 62 | 71 | 68 | 49 |

## Slide 4
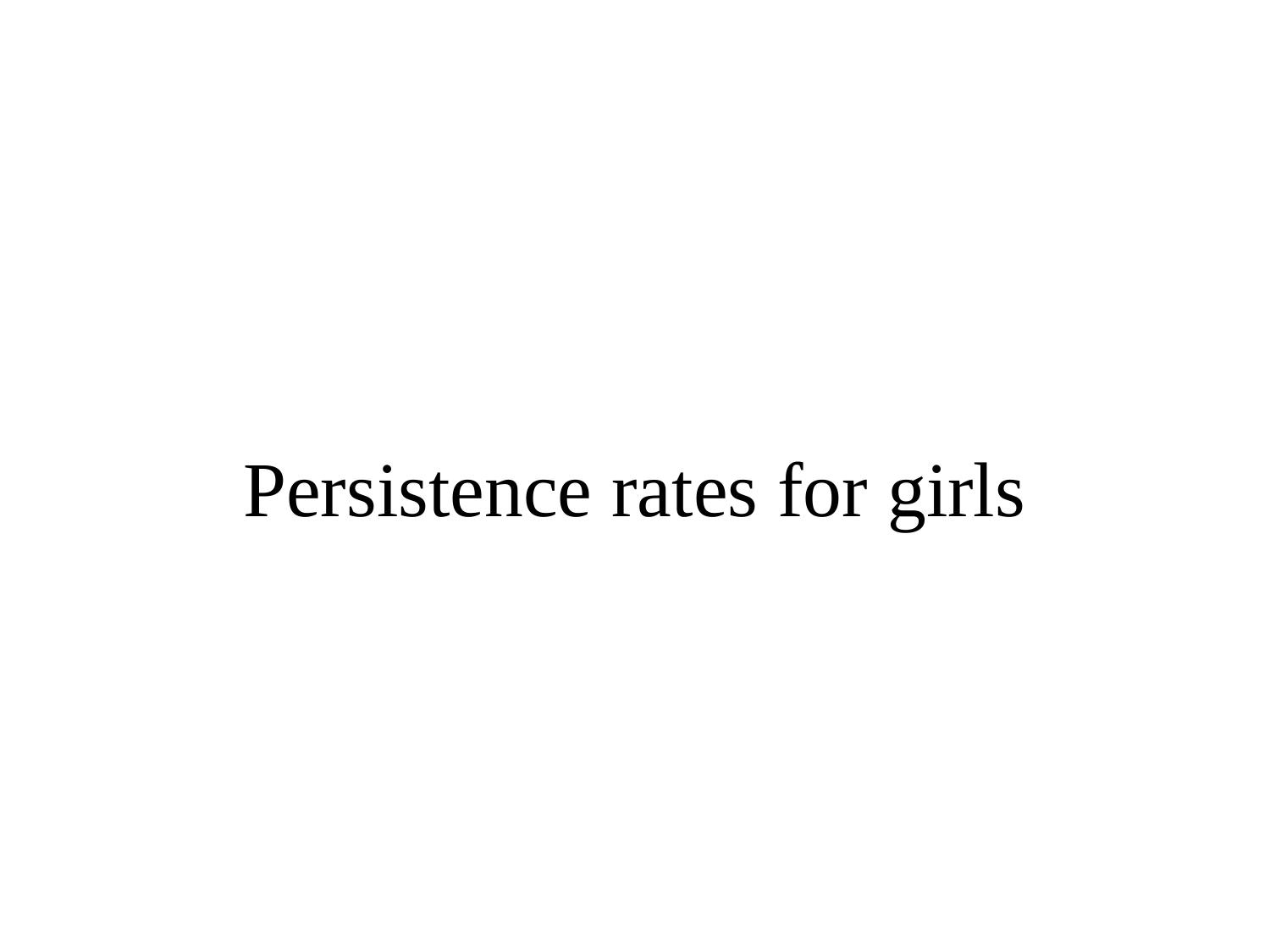

# Persistence rates for girls

## Slide 5
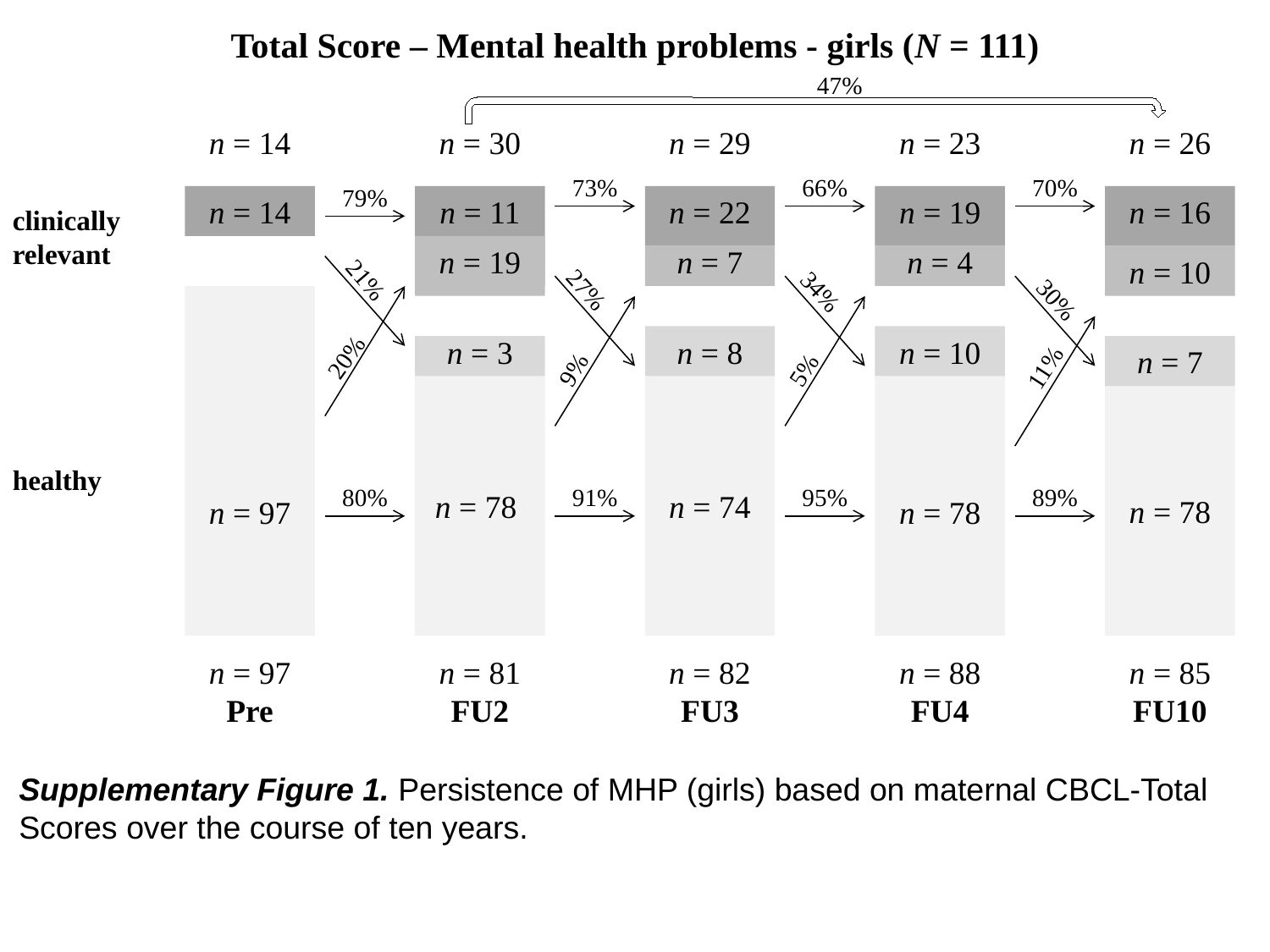

Total Score – Mental health problems - girls (N = 111)
47%
n = 14
n = 30
n = 29
n = 23
n = 26
73%
66%
70%
79%
n = 14
n = 11
n = 22
n = 19
n = 16
clinically relevant
n = 19
n = 7
n = 4
n = 10
21%
27%
34%
30%
n = 3
n = 8
n = 10
n = 7
20%
11%
9%
5%
n = 78
n = 74
n = 78
healthy
80%
91%
95%
89%
n = 97
n = 78
n = 97
Pre
n = 81
FU2
n = 82
FU3
n = 88
FU4
n = 85
FU10
Supplementary Figure 1. Persistence of MHP (girls) based on maternal CBCL-Total Scores over the course of ten years.

## Slide 6
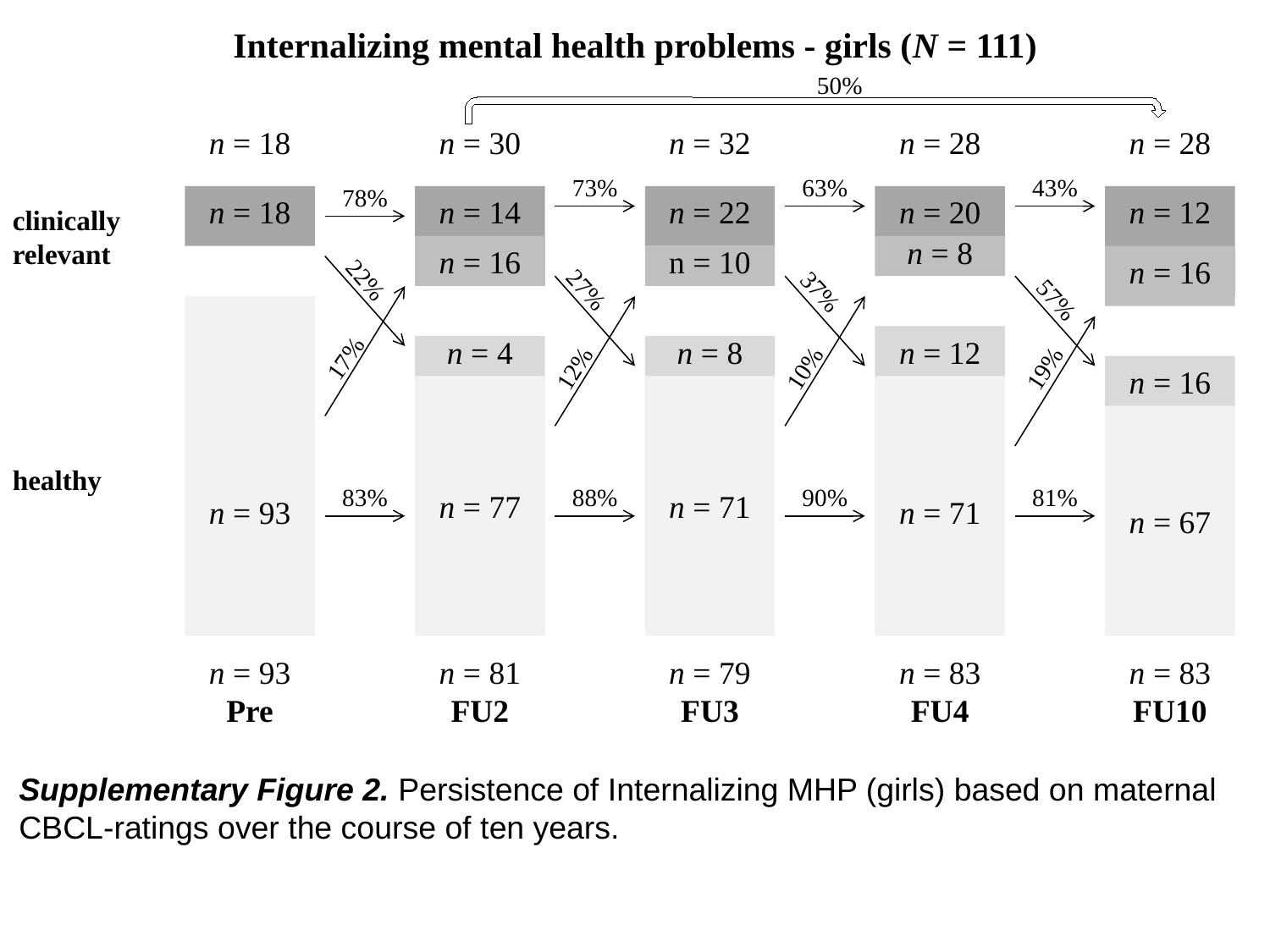

Internalizing mental health problems - girls (N = 111)
50%
n = 18
n = 30
n = 32
n = 28
n = 28
73%
63%
43%
78%
n = 18
n = 14
n = 22
n = 20
n = 12
clinically relevant
n = 8
n = 16
n = 10
n = 16
22%
27%
37%
57%
n = 4
n = 8
n = 12
17%
12%
19%
10%
n = 16
n = 77
n = 71
n = 67
healthy
83%
88%
90%
81%
n = 93
n = 71
n = 93
Pre
n = 81
FU2
n = 79
FU3
n = 83
FU4
n = 83
FU10
Supplementary Figure 2. Persistence of Internalizing MHP (girls) based on maternal CBCL-ratings over the course of ten years.

## Slide 7
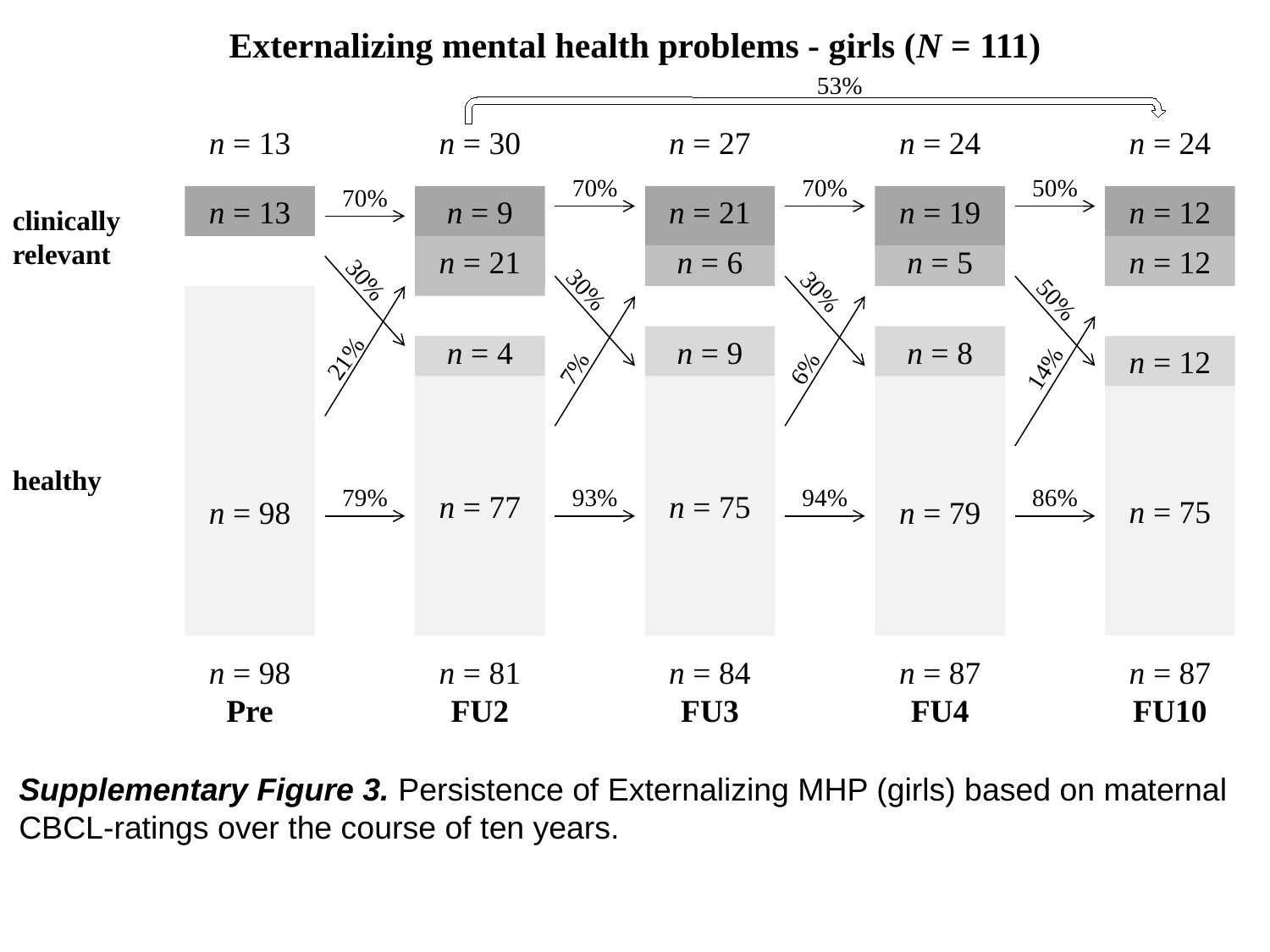

Externalizing mental health problems - girls (N = 111)
53%
n = 13
n = 30
n = 27
n = 24
n = 24
70%
70%
50%
70%
n = 13
n = 9
n = 21
n = 19
n = 12
clinically relevant
n = 21
n = 6
n = 5
n = 12
30%
30%
30%
50%
n = 4
n = 9
n = 8
n = 12
21%
7%
14%
6%
n = 77
n = 75
n = 75
healthy
79%
93%
94%
86%
n = 98
n = 79
n = 98
Pre
n = 81
FU2
n = 84
FU3
n = 87
FU4
n = 87
FU10
Supplementary Figure 3. Persistence of Externalizing MHP (girls) based on maternal CBCL-ratings over the course of ten years.

## Slide 8
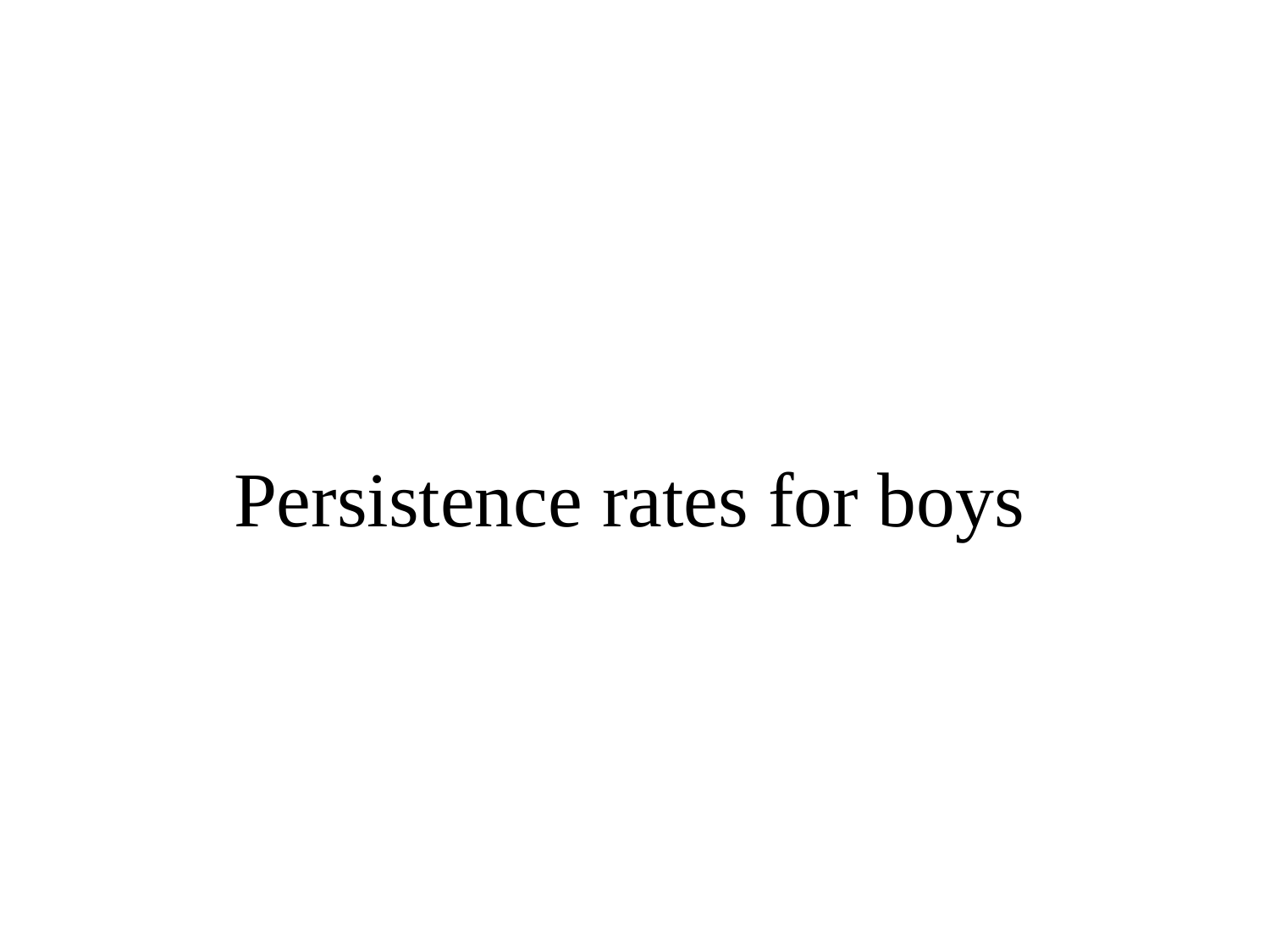

# Persistence rates for boys

## Slide 9
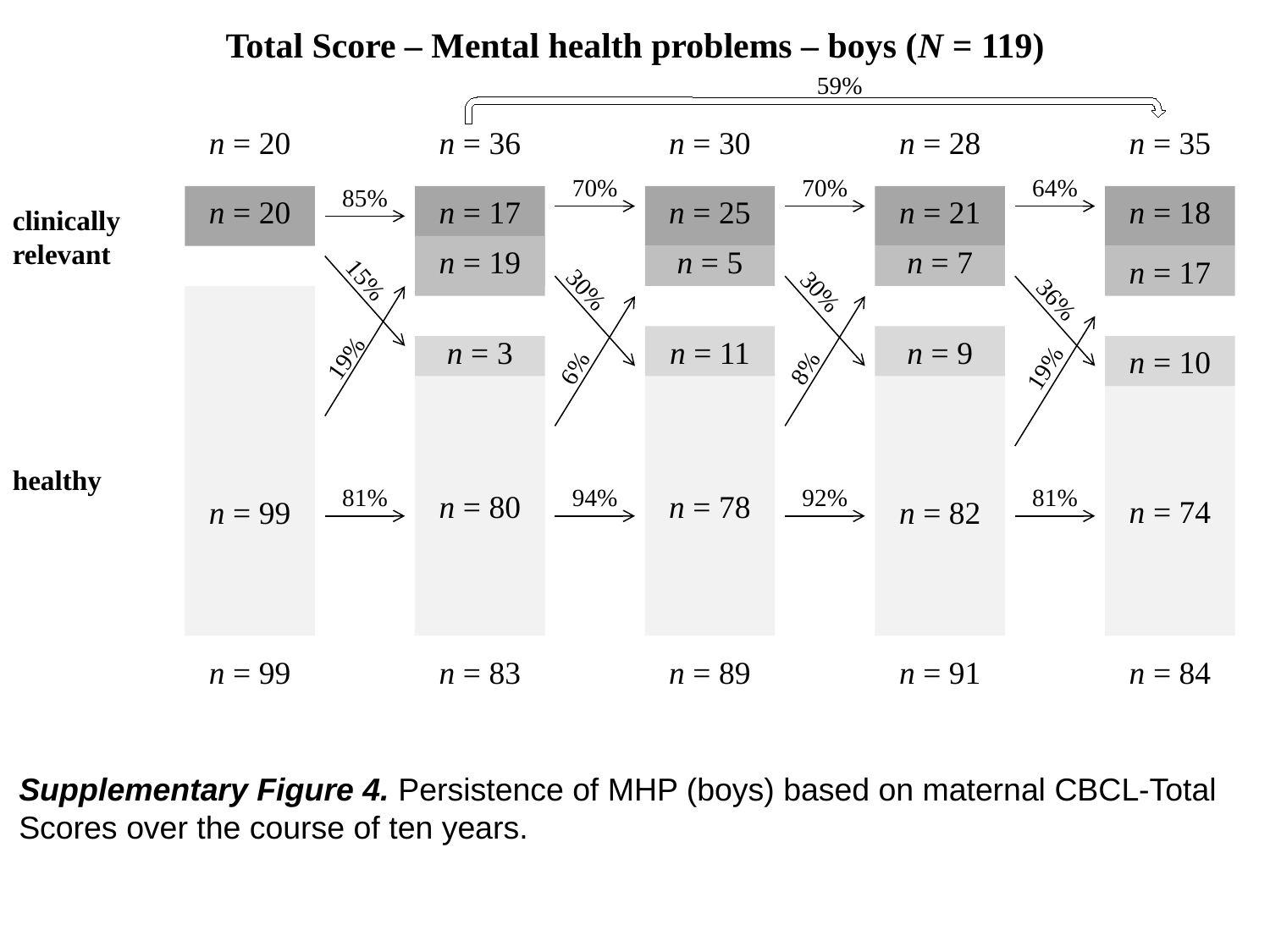

Total Score – Mental health problems – boys (N = 119)
59%
n = 20
n = 36
n = 30
n = 28
n = 35
70%
70%
64%
85%
n = 20
n = 17
n = 25
n = 21
n = 18
clinically relevant
n = 19
n = 5
n = 7
n = 17
15%
30%
30%
36%
n = 3
n = 11
n = 9
n = 10
19%
6%
19%
8%
n = 80
n = 78
n = 74
healthy
81%
94%
92%
81%
n = 99
n = 82
n = 99
n = 83
n = 89
n = 91
n = 84
Supplementary Figure 4. Persistence of MHP (boys) based on maternal CBCL-Total Scores over the course of ten years.

## Slide 10
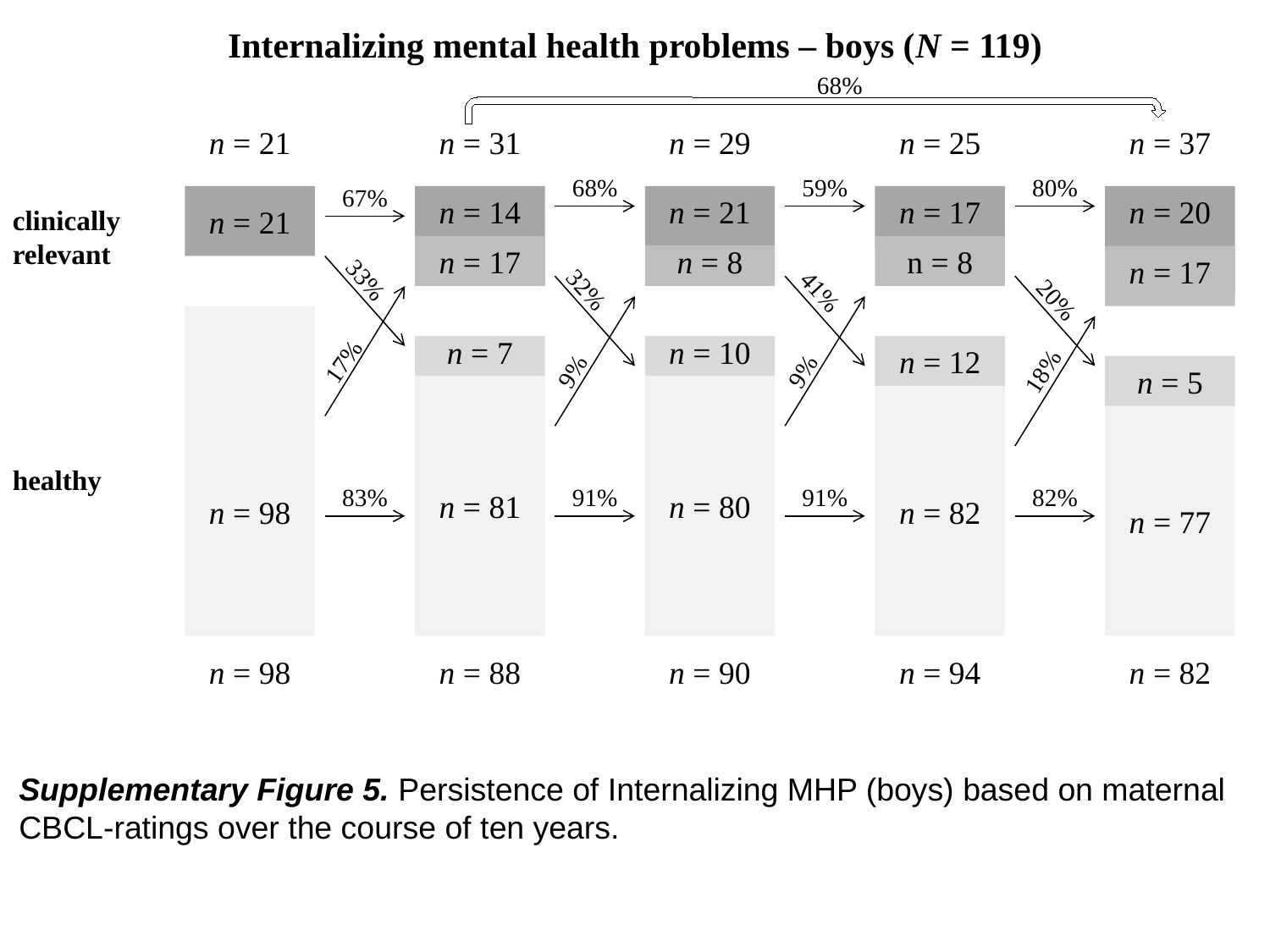

Internalizing mental health problems – boys (N = 119)
68%
n = 21
n = 31
n = 29
n = 25
n = 37
68%
59%
80%
67%
n = 14
n = 21
n = 17
n = 20
clinically relevant
n = 21
n = 17
n = 17
n = 8
n = 8
n = 17
33%
32%
41%
20%
n = 7
n = 10
n = 12
17%
9%
9%
18%
n = 5
n = 81
n = 80
n = 77
healthy
83%
91%
91%
82%
n = 98
n = 82
n = 98
n = 88
n = 90
n = 94
n = 82
Supplementary Figure 5. Persistence of Internalizing MHP (boys) based on maternal CBCL-ratings over the course of ten years.

## Slide 11
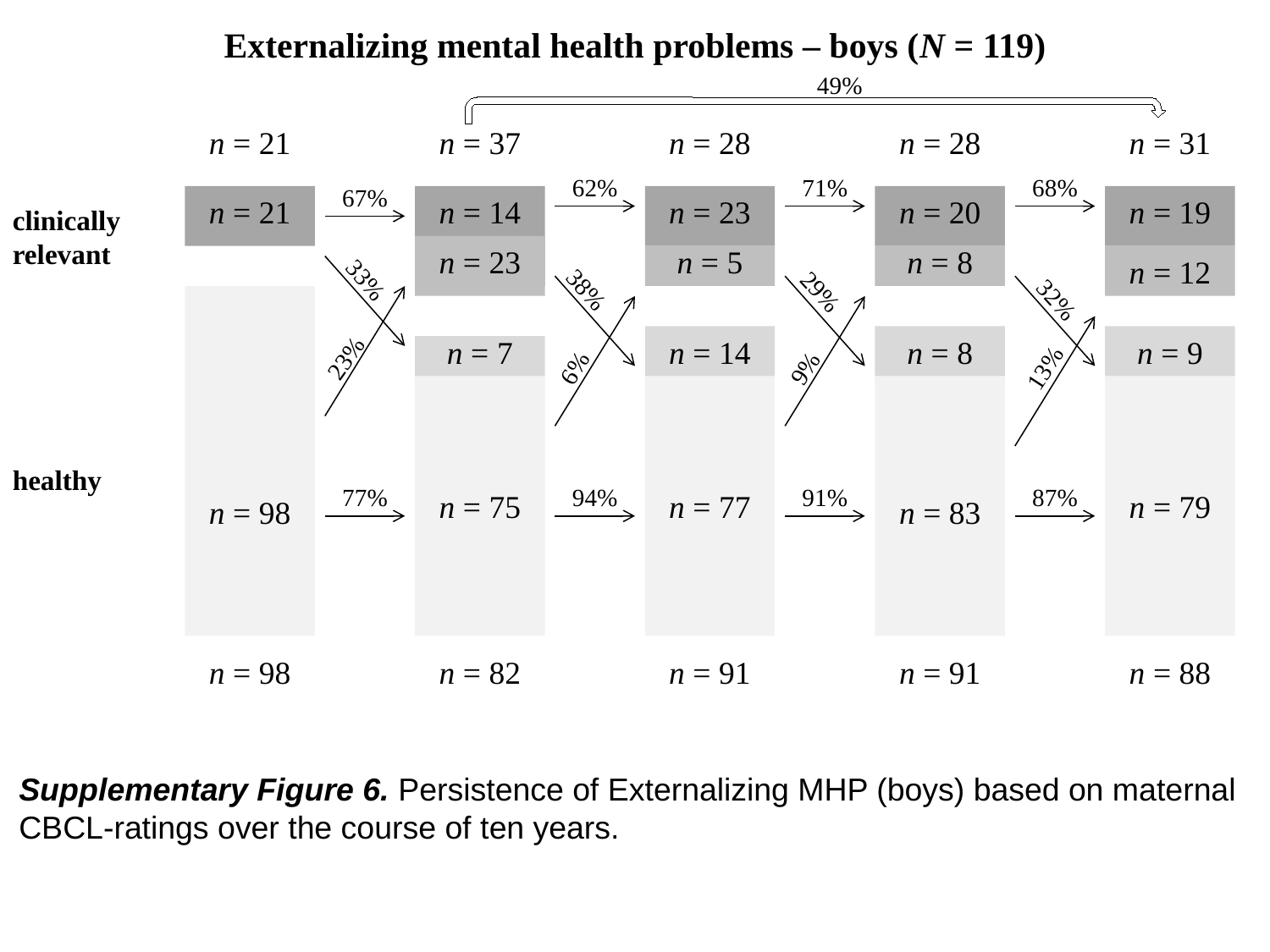

Externalizing mental health problems – boys (N = 119)
49%
n = 21
n = 37
n = 28
n = 28
n = 31
62%
71%
68%
67%
n = 21
n = 14
n = 23
n = 20
n = 19
clinically relevant
n = 23
n = 5
n = 8
n = 12
33%
38%
29%
32%
n = 7
n = 14
n = 8
n = 9
23%
6%
13%
9%
n = 75
n = 77
n = 79
healthy
77%
94%
91%
87%
n = 98
n = 83
n = 98
n = 82
n = 91
n = 91
n = 88
Supplementary Figure 6. Persistence of Externalizing MHP (boys) based on maternal CBCL-ratings over the course of ten years.
